# THE RELATIONSHIP BETWEEN ACTIVE AGING INDEX AND PHYSICAL FUNCTION OF COMMUNITY-DWELLING OLDER ADULTS

**DOI:** 10.1093/geroni/igae098.2960

**Published:** 2024-12-31

**Authors:** Ji Yeong Park, Kyung Hee Lee

**Affiliations:** Yonsei University, Seoul, Republic of Korea; Yonsei University, Seoul, Republic of Korea

## Abstract

Physical function is a key factor for older adults to live longer in their own place maintaining independence. Active Aging Index (AAI) is a multidimensional composite index, that can capture human capacity and enabling environment for active aging. Nevertheless, research rarely addresses the individual-level AAI related to the physical function of older adults living in the community. The aim of this study was to investigate the relationship between AAI and physical function of older adults living in community. We utilized the 2020 Survey of Living Conditions and Welfare Needs of Korean Older Persons, employing complex sampling. The physical function of older adults in the community refer to their ability to perform mobility and self-care duties in their daily life. It was measured using the Physical Functioning Assessment of Community-Dwelling Older Persons, a validated and reliable tool. The AAI, which consisted of 4 Domains (i.e., employment, participation in society, independent, healthy and secure living, and capacity and enabling environment for active aging) and 17 Indicators was entered as explanatory variables. The multiple regressions were performed by complex sampling design. The total active aging index was significantly related to the physical function of older adults living in the community. Three out of four domains (employment, Independent, healthy and secure living, and capacity and enabling environment for active aging) were associated with physical function. The social environment must be considered when establishing policies promoting physical function of the older adults living in the community.

